# Comparison of stage III mucinous and serous ovarian cancer: a case-control study

**DOI:** 10.1186/s13048-018-0464-2

**Published:** 2018-10-30

**Authors:** Zeliha Firat Cuylan, Emine Karabuk, Murat OZ, Ahmet Taner Turan, Mehmet M. Meydanli, Salih Taskin, Mustafa Erkan Sari, Hanifi Sahin, Suat C. Ulukent, Ozgur Akbayir, Kemal Gungorduk, Tayfun Gungor, Mehmet F. Kose, Ali Ayhan

**Affiliations:** 1 Department of Gynecologic Oncology, Zekai Tahir Burak Women’s Health Training and Research Hospital, Faculty of Medicine, University of Health Sciences, Ankara, Turkey; 2 Faculty of Health Sciences, Department of Obstetrics and Gynecology, Acibadem University, Istanbul, Turkey; 3 Department of Gynecologic Oncology, Etlik Zubeyde Hanim Women’s Health Training and Research Hospital, Faculty of Medicine, University of Health Sciences, Ankara, Turkey; 4 Division of Gynecologic Oncology, Department of Obstetrics and Gynecology, School of Medicine, AnkaraUniversity, Ankara, Turkey; 5 Department of General Surgery, Kanuni Sultan Suleyman Teaching and Research Hospital, Faculty of Medicine, University of Health Sciences, Istanbul, Turkey; 6 Department of Gynecologic Oncology, Kanuni Sultan Suleyman Teaching and Research Hospital, Faculty of Medicine, University of Health Sciences, Istanbul, Turkey; 7 Department of Gynecologic Oncology, Tepecik Education and Research Hospital, Faculty of Medicine, University of Health Sciences, Izmir, Turkey; 8 Division of Gynecologic Oncology, Department of Obstetrics and Gynecology, Faculty of Medicine, BaskentUniversity, Ankara, Turkey

**Keywords:** Analyses, survival, Epithelial ovarian cancer, Mucinous adenocarcinoma, Serous cystadenocarcinoma

## Abstract

**Background:**

The purpose of this case-control study was to compare the prognoses of women with stage III mucinous ovarian carcinoma (MOC) who received maximal or optimal cytoreduction followed by paclitaxel plus carboplatin chemotherapy to those of women with stage III serous epithelial ovarian cancer (EOC) treated in the similar manner.

**Methods:**

We performed a multicenter, retrospective review to identify patients with stage III MOC at seven gynecologic oncology departments in Turkey. Eighty-one women with MOC were included. Each case was matched to two women with stage III serous EOC in terms of age, tumor grade, substage of disease, and extent of residual disease. Survival estimates were measured using Kaplan-Meier plots. Variables predictive of outcome were analyzed using Cox regression models.

**Results:**

With a median follow-up of 54 months, the median progression-free survival (PFS) for women with stage III MOC was 18.0 months (95% CI; 13.8–22.1, SE: 2.13) compared to 29.0 months (95% CI; 24.04–33.95, SE: 2.52) in the serous group (*p* = 0.19). The 5-year overall survival rate of the MOC group was significantly lower than that of the serous EOC group (44.9% vs. 66.3%, respectively; *p* < 0.001). For the entire cohort, presence of multiple peritoneal implants (Hazard ratio [HR] 2.39; 95% confidence interval [CI], 1.38–4.14, *p* = 0.002) and mucinous histology (HR 2.28; 95% CI, 1.53–3.40, *p* < 0.001) were identified as independent predictors of decreased OS.

**Conclusion:**

Patients with MOC seem to be 2.3 times more likely to die of their tumors when compared to women with serous EOC.

## Introduction

Primary mucinous ovarian carcinoma (MOC) represents a biochemically and genetically distinct subgroup of epithelial ovarian cancer (EOC) [1]. MOC accounts for 3% [2] to 10% [3] of all ovarian carcinomas. Most patients with MOC are diagnosed at an early stage and therefore it confers a favorable prognosis [3]. However, once the disease is advanced, mucinous histology has a poorer outcome compared to the other histologic subgroups [4–7].

Mucinous histology has consistently been reported as an independent adverse prognostic factor in advanced EOC [6, 8, 9] and the outcomes of MOCs are significantly different from EOCs with serous histology [10]. The reason for poor prognosis of advanced MOC has been suggested to be either the aggressive biology of mucinous tumor or chemoresistance or both [9, 11, 12].

Approximately 14% of all MOCs are diagnosed as stage III disease [7]. Response rates to standard platinum-based chemotherapy are low (12.5–38.5%) among women with MOC, and debulking to minimal or no residual disease (RD) remains the standard of care [13]. It has been reported that overall survival (OS) is 3.8 fold increased in patients who have received optimal cytoreductive surgery (CRS) compared to the patients who have had suboptimal cytoreduction [14].

We wondered whether the worse prognosis associated with mucinous histology is also valid when age, tumor grade, substage of disease, and extent of RD are matched with serous histology in women with stage III EOC who have undergone primary CRS followed by standard intravenous paclitaxel plus carboplatin chemotherapy. Given the low frequency of stage III MOC, a multicenter effort was essential in order to achieve an adequate number in this case-control study. The objective of this retrospective, multicenter study was to compare prognostic factors of women with stage III MOC to those of women with stage III serous EOC treated in the same fashion.

## Methods

After Institutional Review Board approval (IRB Approval Number: 05, Date: November 7th 2017), institutional databases for EOC were utilized to identify eligible cases. The databases consisted of individual patients with EOC who underwent upfront surgery between January 1998 and December 2016 at seven gynecologic oncology departments in Turkey. All patients provided an informed consent at admission for storage of their clinical information and for research use of their clinical data.

The case group consisted of EOC patients with histopathologically confirmed Stage III [15] MOC. We included only the patients with optimal or maximal CRS in the upfront surgery. We excluded the patients who had RD greater than 1 cm. Since the case group included only women having pure mucinous histology; women with mixed histologies including mucinous tumors combined with endometrioid, serous, clear-cell, and/or other histologic types were excluded. The patients who received neoadjuvant chemotherapy, women with synchronous malignancies, and those with incomplete medical records were also excluded from the study.

Using a dependent random sampling method, each case was matched to two patients with serous EOC from a series of 783 women who had undergone maximal or optimal CRS (RD ≤ 1 cm) followed by paclitaxel and carboplatin combination chemotherapy for the same period. In the case and control groups, pairs were matched in terms of age at diagnosis (+/− 10 years), year of diagnosis, grade of the tumor (grade 1, 2, or 3), substage (stage IIIA_1_, IIIA_2_, IIIB, and IIIC), and extent of RD (maximal vs. optimal debulking). While selecting the patients in the control group, we were blinded to the outcomes of the patients in order to prevent possible patient selection bias.

The following clinical data were extracted from patients’ medical, surgical, pathology, and chemotherapy reports: demographic characteristics, preoperative serum cancer antigen 125 (CA 125) levels, type and date of surgical procedure, presence of multiple (≥2) peritoneal implants, ascites status, results of peritoneal washings (negative or positive), appendiceal involvement, omental involvement, size of RD after surgery, stage of disease, time to recurrence, length of follow-up and survival. Pathologic characteristics of the disease were abstracted from original pathology reports. Data were collected from departments with an online standardized form.

Gynecologic oncologists performed all of the operations with the aim of achieving complete cytoreduction. After primary CRS, RD was recorded according to the assessment by the surgical team. Retroperitoneal lymph node dissection was performed after completion of intraabdominal cytoreduction. Number of total lymph nodes (LNs) removed, number of para-aortic LNs removed, and number of pelvic LNs removed and number of metastatic LNs were noted.

All pathologic specimens of the upfront surgery were evaluated and interpreted by expert gynecologic pathologists of the participating institutions with experience in gynecological malignancies but were not reviewed centrally in the current study.MOC was diagnosed after examination of permanent sections. Immunuhistochemical study was performed in women where metastatic tumors could not be excluded. With careful exclusion of noninvasive and metastatic mucinous tumors, women who had final pathologic diagnoses as “primary MOCs” were included in the case group.

The World Health Organization (WHO) criteria were used for histologic classification of the tumors [16, 17]. Architectural grading was defined by standard International Federation of Gynecology and Obstetrics (FIGO) criteria. All tumors were staged according to the FIGO staging system, which was revised in 2014 [15]. In patients treated before 2014, stage was adapted to the FIGO 2014 staging system retrospectively using surgical and pathologic assessment.

All patients (cases and controls) received adjuvant chemotherapy. The standard first line chemotherapy protocol included paclitaxel 175 mg/m^2^ plus carboplatin at the dose of area under curve (AUC) 5 or 6 every 21 days for 6 cycles. Targeted agents (i.e. bevacizumab) were not included in the first line chemotherapy. Platinum refractory disease was defined as disease progression on first-line platinum based regimen, whereas platinum resistant disease included patients with tumor progression within 6 months of completion of platinum based chemotherapy.

We followed-up the patients quarterly for the first 2 years, biannually until 5 years, and annually thereafter. The follow-up visits included physical and gynecologic examinations, and serum CA 125 measurements. Each patient was assessed for relapse with imaging studies and serum CA 125 measurements. Treatment of the recurrences (surgery and/or chemotherapy) was applied according to the institutional practices at that time.

Survival data were last calculated on 31st December 2016. The survival status of the patients was determined as alive or dead at the time of the last follow-up visit. Survival status for all the subjects was confirmed by social security death index search.

Maximal cytoreduction was defined as no visible RD left in-situ (microscopic RD) after primary CRS. Optimal cytoreduction was defined as a RD less than or equal to 1 cm maximal diameter of the largest tumor deposit at the end of the primary surgery. Similarly, suboptimal cytoreduction was defined as > 1 cm of RD. Lymphadenectomy was defined as the dissection of pelvic and para-aortic LN’s at the same time. Progression-free survival (PFS) was defined as the time, in months, from the primary surgery to the documented recurrence with clinical examination and/or radiologic imaging; or death from any cause, whichever occurred first, or the date of last follow-up for patients remaining alive without recurrence. Patients who had no active disease at the last contact were censored in the PFS analysis. Overall survival (OS) was defined as the time, in months, between the date of primary surgery to the date of death or the last follow-up. Surviving patients were censored at their last known follow-up.

Survival curves were generated using Kaplan-Meier plots and survivals were compared using the log-rank test. The chi-squared test was used for nominal variables. For continuous variables, Student’s *t*-test and the Mann-Whitney *U* tests were used for continuous variables with and without normal distribution, respectively. Cox logistic regression models were used to determine co-variates affecting survival, and presented as hazard ratios (HRs) with 95% confidence interval (CI), unadjusted or adjusted for all factors. All variables with a *p* value < 0.05 in the univariate analysis were included in the multivariate analysis. All statistical analyses were performed with the SPSS software version 23.0 (IBM Corp., Armonk, NY, USA). A *p* value < 0.05 was considered statistically significant.

## Results

One hundred and sixty-four women were identified with a postoperative pathology-proven diagnosis of stage III primary MOC at seven participating centers during the study period. We excluded 74 women who had suboptimal CRS, six women who received neoadjuvant chemotherapy, one with synchronous breast cancer and two women with incomplete medical records. Therefore, 81 women with stage III MOC were included in the final analysis. These 81cases were compared to 162 controls with serous EOC who had maximal or optimal CRS followed by paclitaxel plus carboplatin combination chemotherapy.

Table 1 demonstrates the demographic and clinicopathologic characteristics of the study population. Age, menopausal status, presence of multiple peritoneal implants, tumor grade, substage of disease, positive peritoneal cytology, extent of RD, appendiceal involvement, and omental involvement were similar between the cases and controls. Patients with serous EOC were more likely to have elevated median baseline serum CA 125 levels (514 IU/ml vs. 126 IU/ml; *p* = 0.022), ascites (125/162 vs. 42/81; *p* < 0.001), and positive retroperitoneal LNs (116/162 vs. 21/81; *p* < 0.001). The median number of total LNs harvested, the median number of pelvic LNs removed were significantly higher in the serous EOC patients when compared to women with MOC as well as the median number of para-aortic LNs removed (49 vs. 34; *p* < 0.001, 32 vs. 24; *p* = 0.003, and 15 vs. 11; *p* < 0.001, respectively). In both of the groups, stage IIIA_1_, IIIA_2_, IIIB and IIIC disease were noted in 16.1%, 7.4%, 16.0% and 60.5%, respectively.

**Table 1:** Demographic and clinicopathologic characteristics of the study population (*n* = 243)

Characteristics	MOC	Serous EOC	
	Values, n (%)	Values, n (%)	*p*
Age, y (median, range)	53.9 (18–77)	53.4 (28–81)	0.73
Menopausal status			
Postmenopausal	49 (60.5%)	98 (60.5%)	1.0
Premenopausal	32 (39.5%)	64 (39.5%)	1.0
CA 125 (IU/ml) (median, range)	126 (4–4891)	514 (8–27,580)	0.022
Grade			
1	29 (35.8%)	58 (35.8%)	1.0
2–3	52 (64.2%)	104 (64.2%)	1.0
Stage			
IIIA1	13 (16.1%)	26 (16.1%)	1.0
IIIA2	6 (7.4%)	12 (7.4%)	1.0
IIIB	13 (16%)	26 (16%)	1.0
IIIC	49 (60.5%)	98 (60.5%)	1.0
Debulking			
Optimal	54 (66.7%)	108 (66.7%)	1.0
Maximal	27 (33.3%)	54 (33.3%)	1.0
Ascites			
Present	42 (51.9%)	125 (77.2%)	< 0.001
Absent	39 (48.1%)	37 (22.8%)	
Peritoneal Cytology			
Positive	61 (75.3%)	137 (84.6%)	0.114
Negative	20 (24.7%)	25 (15.4%)	
Number of LNs removed (median, range)	34 (19–96)	49 (21–203)	< 0.001
Pelvic LNs	24 (10–75)	32 (10–101)	0.003
Para-aortic LNs	11 (5–54)	15 (5–102)	< 0.001
Retroperitoneal LN metastases			
Present	21 (25.9%)	91 (56.2%)	< 0.001
Absent	60 (74.1%)	71 (43.8%)	
Appendiceal Involvement			
Present	29 (35.8%)	59 (36.4%)	1.0
Absent	52 (64.2%)	103 (63.6%)	
Omental Involvement			
Present	61 (75.3%)	113 (69.8%)	0.451
Absent	20 (24.7%)	49 (30.2%)	
Peritoneal Involvement			
Present	57 (70.4%)	103 (63.6%)	0.318
Absent	24 (29.6%)	59 (36.4%)	
Status			
Alive	32 (39.5%)	112 (69.1%)	
Dead	49 (60.5%)	50 (30.9%)	
Follow-up, months (median, range)	21 (1–216)	46 (1–121)	

Abbreviations: *n* Number, *LN* Lymph node, *y* year, *MOC* Mucinous Ovarian Carcinoma, *EOC* Epithelial Ovarian Carcinoma, *SD* Standard Deviation

**Fig. 1:**
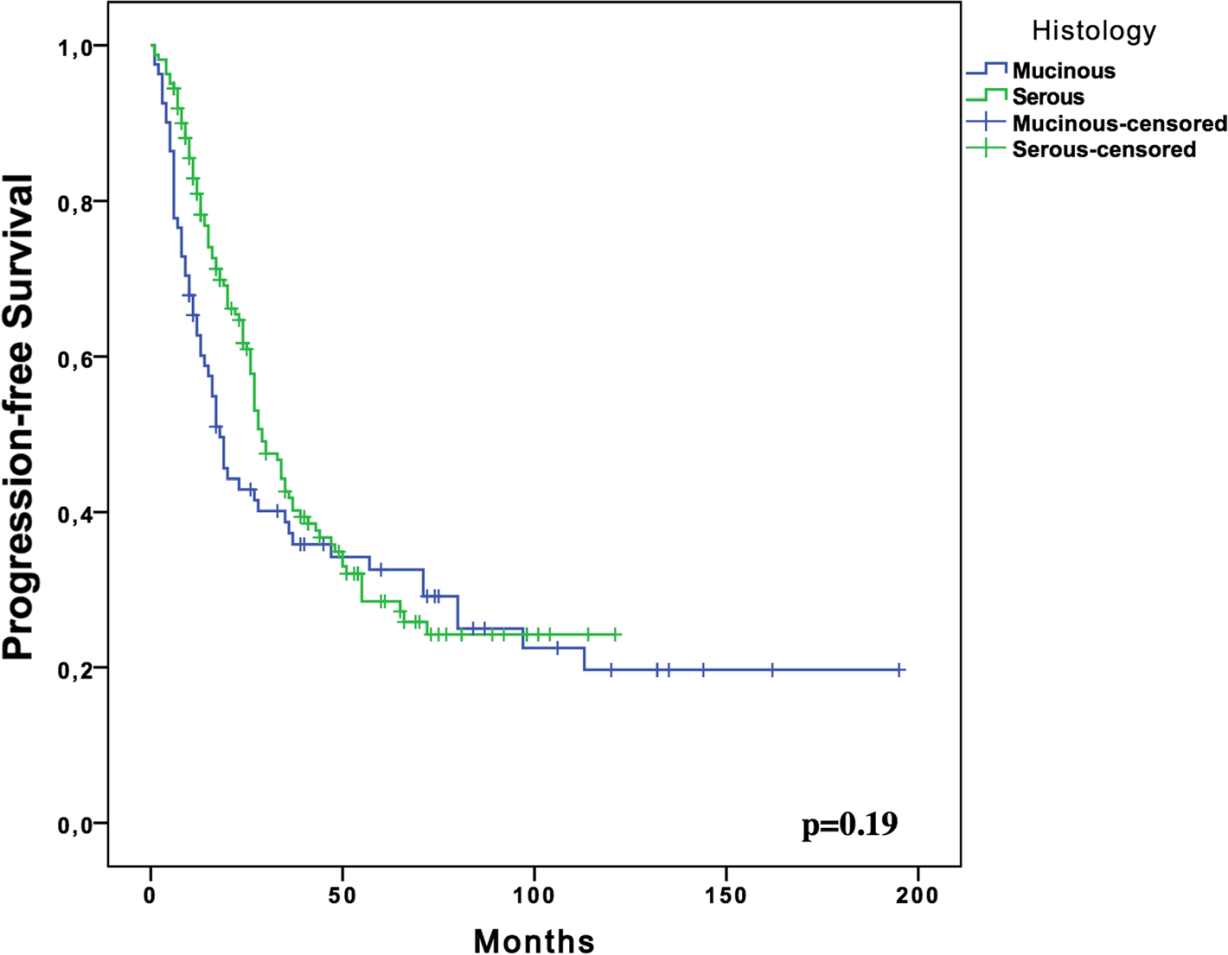
Kaplan Meier Plots for progression free survival of the women with Stage III mucinous (*n* = 81) and serous (*n* = 162) ovarian cancer

**Fig. 2:**
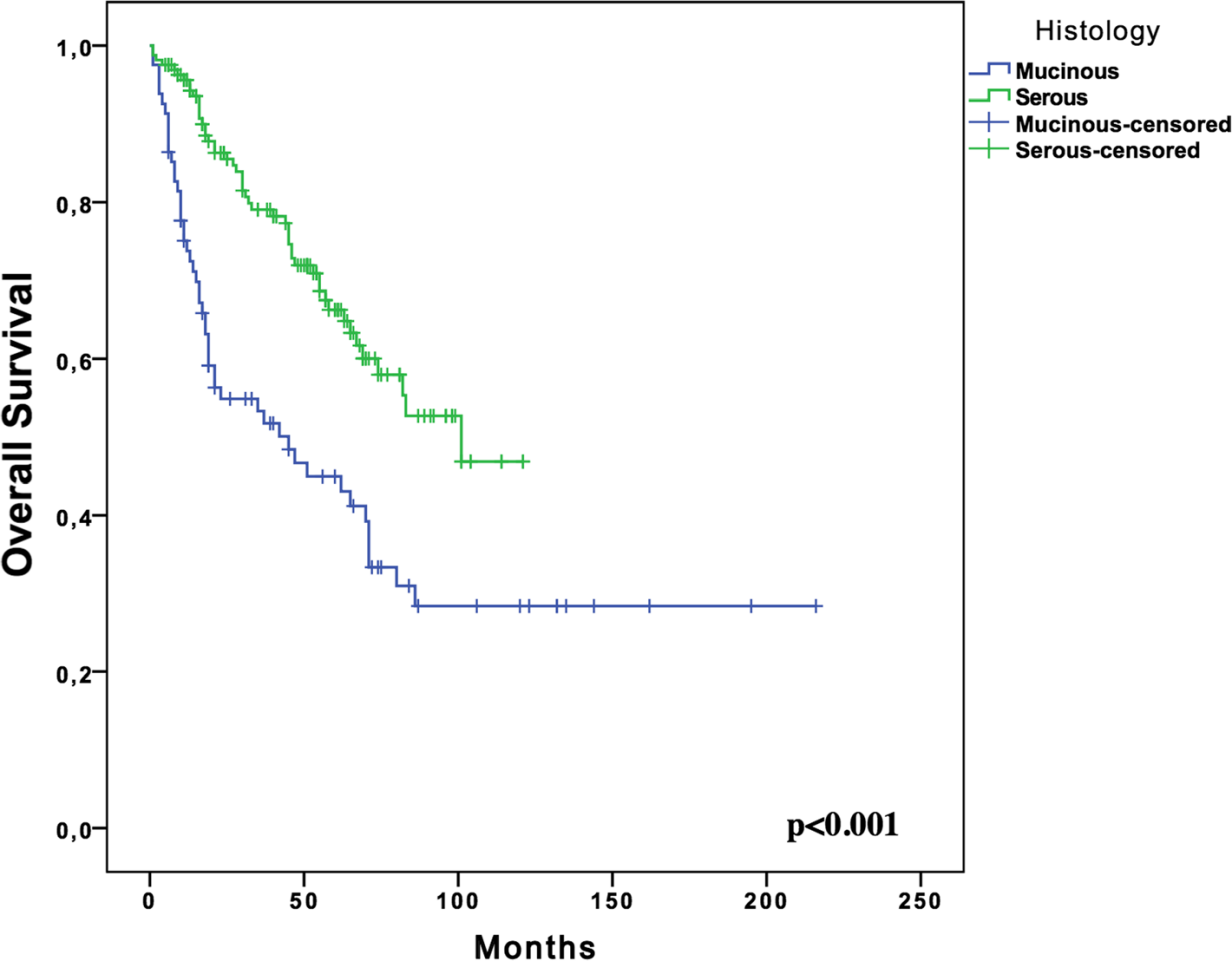
Kaplan Meier Plots for overall survival of the women with Stage III mucinous (*n* = 81) and serous (*n* = 162) ovarian cancer

With a median follow-up of 54 months (range, 5–216 months), the median PFS for women with stage III MOC was 18.0 months (95% CI; 13.8–22.1, SE: 2.13) compared to 29.0 months (95% CI; 24.04–33.95, SE: 2.52) in the serous EOC group (*p* = 0.19) (Fig. 1). The 5-year OS rate of the MOC group was significantly lower than that of the serous EOC group (44.9% vs. 66.3%, respectively; *p* < 0.001) (Fig. 2).

Platinum refractory disease was detected more frequently in the mucinous group (*n* = 18, 22.2%) than in the serous group (*n* = 9, 5.5%) (*p* < 0.001). However, the number of women with platinum resistant disease was comparable between the mucinous and serous groups (*n* = 12 [14.8%] and *n* = 21 [12.9%], respectively, *p* = 0.69).

For the entire cohort, univariate analysis revealed substage of disease (stage IIIA_1_ vs. other stages) (*p* < 0.001), positive peritoneal cytology (*p* = 0.01), appendiceal involvement (*p* < 0.001), omental involvement (*p* < 0.001), and presence of multiple peritoneal implants (*p* < 0.001) as significant factors for decreased PFS. In multivariate analysis, presence of multiple peritoneal implants (HR 2.1, 95% CI 1.40–3.14; *p* < 0.001) remained as the sole independent risk factor for decreased PFS.

For the entire cohort, univariate analysis revealed substage of disease (stage IIIA_1_ vs. other stages) (*p* = 0.001), appendiceal involvement (*p* < 0.001), omental involvement (*p* < 0.001), presence of multiple peritoneal implants (*p* < 0.001), and mucinous histology (*p* < 0.001) as significant factors for decreased OS (Table 2). Multivariate analysis demonstrated presence of multiple peritoneal implants (HR 2.39; 95% CI, 1.38–4.14, *p* = 0.002) and mucinous histology (HR 2.28; 95% CI, 1.53–3.40, *p* < 0.001) as independent predictors of decreased OS (Table 2).

**Table 2:** Univariate and multivariate analyses for overall survival in the entire cohort (stage III mucinous [*n* = 81] and serous [*n* = 162] ovarian carcinoma)

	Univariate analysis OS^a^ N of events (%)	*p*	Multivariateanalysis		
			HR	CI 95%	*p*
Age, y					
< 52	36/118 (61.7%)	0.163			
≥ 52	47/125 (56.7%)				
Menopausal Status					
Premenopausal	33/96 (56.6%)	0.89			
Postmenopausal	50/147 (60.5%)				
Ca-125(IU/ml)					
< 310	46/121 (56.2%)	0.089			
≥ 310	37/122 (62.2%)				
Grade					
1	33/87 (55.1%)	0.74			
2–3	50/156 (61.5%)				
Ascites					
Present	62/167 (56.2%)	0.371			
Absent	21/76 (66%)				
Retroperitoneal LN metastases					
Present	44/112 (39.2%)	0.646			
Absent	55/131 (41.9%)				
Peritoneal cytology					
Positive	73/198 (56.5%)	0.215			
Negative	10/45 (71.8%)				
Stage					
IIIA1	6/39 (78.4%)	0.001			
IIIA2, IIIB, IIIC	77/204 (55.6%)				
Appendiceal Involvement					
Present	41/88 (45%)	< 0.001			
Absent	42/155 (66.9%)				
Omental Involvement					
Present	71/174 (52%)	< 0.001			
Absent	12/69 (77.6%)				
Peritoneal Involvement					
Present	67/160 (49.2%)	< 0.001	2.39	1.38–4.14	0.002
Absent	16/83 (76.9%)				
Histologic Subtype					
Mucinous	41/81 (44.9%)	< 0.001	2.28	1.53–3.40	< 0.001
Serous	42/162 (66.3%)				

^a^**:**5-year overall survival rate

Abbreviations: *OS* Overall Survival, *LN* Lymph node, *HR* Hazard ratio, *CI* Confidence interval, *y* Year, *N* number

Finally, 81 patients with stage III MOC were separately analyzed. Univariate analysis revealed substage of disease (stage IIIA_1_ vs. other stages) (*p* < 0.001), omental involvement (*p* = 0.004), and optimal debulking (*p* < 0.001) as significant factors for decreased PFS. In the multivariate Cox regression model, only maximal CRS (HR 0.48; 95% CI, 0.23–0.97, *p* = 0.041) was identified as an independent predictor of increased PFS. For women with stage III MOC, univariate analysis revealed substage of disease (stage IIIA_1_ vs. other stages) (*p* = 0.002), omental involvement (*p* = 0.008), and optimal debulking (*p* < 0.001) as significant factors for decreased OS (Table 3). In multivariate analysis, only maximal CRS (HR 0.37; 95% CI, 0.16–0.86, *p* = 0.021) remained as an independent predictor of increased OS (Table 3). At the time of reporting, of 81 women with stage III MOC, 49 (60.5%) were dead whereas 32 (39.5%) were alive. The corresponding figures were found to be 50 (30.9%) and 112 (69.1%), respectively in the serous EOC group.

**Table 3:** Univariate and multivariate analyses for overall survival in women with stage III mucinous ovarian carcinoma (*n* = 81)

	Univariate analysis OS^a^ N of events (%)	*p*	Multivariate analysis		
			HR	CI 95%	*p*
Age, y					
< 54	17/39 (52.2%)	0.126			
≥ 54	24/42 (38.1%)				
Menopausal Status					
Premenopausal	15/32 (49%)	0.49			
Postmenopausal	26/49 (42%)				
Ca-125(IU/ml)					
< 126	19/40 (49.2%)	0.42			
≥ 126	22/41 (40.4%)				
Grade					
1	16/29 (36%)	0.98			
2–3	25/52 (48%)				
Ascites					
Present	24/42 (37.4%)	0.174			
Absent	17/39 (52.4%)				
LN metastases					
Present	13/21 (27.9%)	0.433			
Absent	28/60 (50.4%)				
Peritoneal cytology					
Positive	35/61 (36.8%)	0.092			
Negative	6/20 (67.7%)				
Appendiceal Involvement					
Present	18/29 (30.9%)	0.180			
Absent	23/52 (51.9%)				
Omental Involvement					
Present	35/61 (37%)	0.008			
Absent	6/20 (68.4%)				
Stage					
IIIA1	2/13 (83.9%)	0.002			
IIIA2, IIIB, IIIC	39/68 (37%)				
Debulking					
Maximal	6/27 (74.3%)	< 0.001	0.37	0.16–0.86	0.021
Optimal	35/54 (30.1%)				

^a^**:**5-year overall survival rate

Abbreviations: *MOC* Mucinous Ovarian Carcinoma, *OS* Overall Survival, *LN* Lymph node, *HR* Hazard ratio, *CI* Confidence interval, *y* Years

## Discussion

Our results indicate that presence of multiple peritoneal implants and mucinous histology seem to be the independent predictors of decreased OS in a cohort of 243 women who have undergone maximal or optimal CRS and found to have either mucinous or serous stage III EOC at the end of final pathology report. Patients with MOC were 2.3 times more likely to die of their tumors when compared to women with serous EOC.

The retrospective nature of the study and lack of central pathology review seem to be the major limitations of the current study. Patients were treated and observed at seven different gynecologic cancer centers. Therefore, we might not have presented the clinical outcomes of the recurrent patients objectively and equally since the treatment of recurrences was not uniform, and changed according to the institutional practices at that time.

It has been reported that the clinical behavior of MOC is distinctly different from serous EOC and the prognosis of MOC is worse when compared to other histologies [6–9, 14, 18–21]. Hess et al. [18] have reported that MOC has a HR of 2.94 for progression and a HR of 3.08 for death in a study of 27 women with stage III/IV MOC. Winter et al. [19] reported that mucinous histology was associated with a worse PFS and OS compared with serous carcinomas in a study including 34 women with stage III MOC. In that study, the median PFS was 10.5 months whereas the median OS was 14.8 months for women with MOC.

In a meta-analysis consisting of 264 women with stage III/IV MOC, Mackay et al. [20] reported that patients with MOC had an increased risk of death (HR 2.66, 95% CI 2.29–3.08) and disease-progression (HR 2.10, 95% CI 1.84–2.41) compared to those with serous carcinoma. Patients with mucinous histology had an estimated median PFS of 7.6 months and a median OS of 14.6 months in that meta-analysis. Bamias et al. [21] reported the median OS as 15.0 months for 21 women with stage III/IV MOC compared to 45.0 months for 389 women with stage III/IV ovarian high-grade serous carcinoma. In a case-control study, Karabuk et al. [14] have reported the median PFS and OS as 7.0 and 35.0 months, respectively, for 50 women with stage III/IV MOC. The authors concluded that the risk of death for MOC patients was significantly higher than that of serous EOC patients (HR 2.14, 95% CI 1.34–3.42). In the current study, we have found out the median PFS and OS as 18.0 and 45.0 months, respectively, for women with stage III MOC who have undergone maximal or optimal CRS. The corresponding figures were 29.0 and 101.0 months, respectively, for the serous counterpart. There was no significant difference between the cases and the controls in terms of PFS. However, women with MOC were 2.3 times more likely to die of their tumors when compared to patients with serous EOC in the current study. Table 4 compares the findings of the current study with those of previous studies.

**Table 4:** Comparison of the findings of the current study with those of previous studies associated with advanced mucinous ovarian carcinoma

Author	Number of women with MOC	Stage of disease	Extent of residual disease	Median PFS (months)	Media OS (months)
Hess, 2004 [18]	27	III, IV	optimal or suboptimal	5.7	12.0
Pectasides, 2005 [27]	47	III, IV	optimal or suboptimal	11.8	33.2
Winter, 2007 [19]	34	III	optimal or suboptimal	10.5	14.8
Bamias, 2010 [8]	24	III, IV	optimal or suboptimal	NR	15.4
Mackay, 2010 [20]	264	III, IV	optimal or suboptimal	7.6	14.6
Bamias, 2012 [21]	21	III, IV	optimal or suboptimal	NR	15.0
Karabuk, 2013 [14]	50	III, IV	optimal or suboptimal	7.0	35.0
Current study	81	III	maximal or optimal	18.0	45.0

Abbreviations: *MOC* mucinous ovarian carcinoma, *PFS* progression-free survival, *OS* overall survival, *NR* not reported

Using the Surveillance, Epidemiology and End Results (SEER) database, Schiavone et al. [7] reported that the HR for OS was 1.60 for women with stage III MOC. The 5-year survival rate for women with stage III serous EOC was 33.6% compared with 25.7% for MOC. The corresponding rates were 66.3%, and 44.9%, respectively, in our study. It should be emphasized that the SEER database lacks several important variables such as extent and outcome of initial surgery including the size of RD, type of adjuvant chemotherapy, and recurrence. However, all patients (cases and controls) in the current study underwent maximal or optimal CRS followed by paclitaxel/carboplatin chemotherapy and cases were matched to controls in terms of age, tumor grade, substage of disease, and extent of RD.

Favorable prognostic factors such as younger patient age, lower tumor grade, and less peritoneal carcinomatosis have been reported for women with MOC [9, 22]. However, maximal cytoreduction was identified as the sole independent prognostic factor for increased PFS and OS in the current study. The volume of tumor left after primary CRS depends on the number and the size of the tumor elements [5]. We were not able to define the number of lesions left after initial surgery in this retrospective analysis. The maximal diameter of the largest residual tumor nodule is a very crude estimate of RD [5, 23], but it still gave valuable prognostic information in our study.

Melamed et al. [13] have recently reported that cytoreduction to no gross RD is associated with a hazard reduction of 54% compared to any gross RD in MOCs.. The optimal chemotherapy regimen for MOC has not been clarified yet; so aggressive CRS seems to be the only effective treatment to improve the prognosis in advanced-stage MOC [12]. Our finding associated with maximal cytoreduction as the sole independent prognostic factor for both PFS and OS in women with stage III MOC is in agreement with previous reports [12, 13].

The most important limitation of the current study is the fact that central pathology review was not performed. There is now increasing recognition that many MOCs may in fact be metastatic mucinous neoplasms from other primary sites [7]. Differentiating MOC from gastrointestinal cancer [24, 25], mainly carcinomas of appendix, and colorectal cancer by morphology alone can be difficult. It should be reminded that no patients receiving neoadjuvant chemotherapy were included in the current study and all patients have undergone surgical exploration which commonly would have identified a cancer of gastrointestinal origin. Additionally, immunohistohemical studies were performed in cases where metastatic tumors could not be excluded. Recent data suggests that the application of updated criteria results in reliable histopathological diagnoses based on the cell type [26]. Nevertheless, our study was restricted by the lack of a central pathology review as many previous reports [7, 13, 20, 27].

The major strength of the current study is the inclusion of a relatively large number of patients with stage III MOC. Our study has the advantage that all included patients were treated with the standard paclitaxel/carboplatin regimen. The standard surgery and the standard postoperative adjuvant chemotherapy regimen seem to improve the reliability of our findings and decrease the probable effects of the confounders.

We conclude that women with stage III MOC seem to be 2.3 times more likely to die of their tumors when compared to patients with serous EOC. Maximal cytoreduction seems to be the sole independent prognostic factor for increased OS in women with stage III MOC. As the extent of RD is a modifiable prognostic factor, it seems reasonable to perform maximal CRS whenever possible in order to improve the outcomes of those women.
